# Constitutive and induced insect resistance in RNAi-mediated ultra-low gossypol cottonseed cotton

**DOI:** 10.1186/s12870-019-1921-9

**Published:** 2019-07-18

**Authors:** Steffen Hagenbucher, Michael Eisenring, Michael Meissle, Keerti S. Rathore, Jörg Romeis

**Affiliations:** 1 Agroscope, Research Division Agroecology and Environment, Reckenholzstrasse 191, 8046 Zürich, Switzerland; 2 Department of Soil and Crop Sciences, Institute for Plant Genomics & Biotechnology, Texas A&M University, College Station, TX USA

**Keywords:** ULGCS, Glandless cotton, *Gossypium hirsutum*, Gossypol, *Spodoptera littoralis*, Genetically modified crops, TAM66274

## Abstract

**Background:**

Besides fibers, cotton plants also produce a large amount of seeds with a high oil and protein content. The use of these seeds is restricted by their high contents of the terpenoid gossypol, which is harmful to humans and livestock. Using a genetic engineering approach, “Ultra-low gossypol cottonseed” (ULGCS) plants were produced by knocking down an enzyme that catalyzes the formation of a precursor of gossypol. This was accomplished via RNAi-mediated silencing of the target gene using a seed-specific *α-globulin* promotor. Since gossypol is also a crucial defense mechanism against leaf-feeding herbivores, ULGCS plants might possess lower herbivore resistance than non-engineered plants. Therefore, we tested the constitutive and inducible direct insect resistance of two ULGCS cotton lines against the African cotton leafworm, *Spodoptera littoralis*.

**Result:**

The herbivore was equally affected by both ULGCS lines and the control (Coker 312) line when feeding on fully expanded true leaves from undamaged plants and plants induced by jasmonic acid. When plants were induced by caterpillar-damage, however, *S. littoralis* larvae performed better on the ULGCS plants. Terpenoid analyses revealed that the ULGCS lines were equally inducible as the control plants. Levels of terpenoids were always lower in one of the two lines. In the case of cotyledons, caterpillars performed better on ULGCS cotton than on conventional cotton. This was likely caused by reduced levels of gossypol in ULGCS cotyledons.

**Conclusion:**

Despite those effects, the insect resistance of ULGSC cotton can be considered as largely intact and the plants may, therefore, be an interesting alternative to conventional cotton varieties.

**Electronic supplementary material:**

The online version of this article (10.1186/s12870-019-1921-9) contains supplementary material, which is available to authorized users.

## Background

Cotton plants from the genus *Gossypium* are one of the major sources of fiber. Today 95% of all cotton are derived from *Gossypium hirsutum* (Malvaceae: Malvales) [1]. Besides its fibers, cotton plants also produce a large amount of seeds (1.65 kg seeds per kg lint) [2]. The seeds are rich in protein and are a valuable source of oil and fodder [3, 4]. However, they typically contain high concentrations of the terpenoid gossypol, present within the glands of seed kernels. Most of the other aerial parts of cotton plants also have subepidermal glands and those in the green parts of the plant contain not just gossypol, but also hemigossypolone and heliocides 1–4, all derived from the same biosynthetic pathway. Gossypol is known to be toxic to non-ruminant animals [5]. Therefore, a major goal in cotton breeding has been to select for plants that do not produce gossypol. Breeders managed to achieve this goal by producing so-called glandless cotton [2]. However, gossypol and related terpenoids are important herbivore and pathogen resistance factors which are inducible by leaf-chewing herbivores or the direct application of the phytohormone jasmonic acid, a key regulator of cotton defense responses [6]. Glandless cotton is therefore more susceptible to insect pests and diseases [6, 7]. Therefore, there is a need for cotton plants that produce seeds with low levels of gossypol, but unchanged amounts of terpenoids in other plant parts. Using a genetic engineering approach, such “Ultra-low gossypol cottonseed” (ULGCS) plants were produced by knocking down the production of δ-cadinene synthase, an enzyme that catalyzes the formation of δ-cadinene, a precursor of gossypol. This was accomplished via RNAi-mediated silencing of the target gene using a seed-specific *α-globulin* promotor [8, 9]. One transformation event TAM66274 (line 66–274 in our manuscript) has been approved by US regulators in October 2018 [10]).

One concern related to these ULGCS plants is that they could have an increased susceptibility to herbivores and/or diseases. Previous work on induced terpenoid production has focused on salicylic acid driven resistance against diseases [11], and no data exists on the jasmonic acid driven induction pathways that are commonly associated with damage from foliage and fruit-feeding pests of cotton such as *Heliothis*, *Helicoverpa* and *Spodoptera* species (all Lepidoptera: Noctuidae). As cotton terpenoids are important for cotton insect resistance [6, 7], changes in this pathway could have significant effects on plant health and ultimately yield. Although Rathore et al. [11] reported that the constitutive terpenoid production in true leaves is not impaired in ULGCS lines, it is greatly reduced in the cotyledons. Therefore, a lack of gossypol in the cotyledons of ULGCS cotton could make seedlings of these plants more sensitive to herbivore attacks than conventional plants. While a previous study did show that the response of ULGCS cotton against the fungal pathogen *Rhizoctonia solani* (Cantharellales: Ceratobasidiaceae) is intact [11], no data have yet been collected on insect resistance.

In this study, we tested the strength of constitutive and induced insect resistance in ULGCS cotton using two different transgenic events and their non-transformed parental cv. Coker 312. We tested the performance of the generalist herbivore *Spodoptera littoralis* (Lepidoptera: Noctuidae) as a model species. The moth is a multivoltine, polyphagous herbivore found in Africa, the Middle East and the Mediterranean area and is known to attack at least 130 different plants species in 56 families [12]. *Spodoptera littoralis* is considered an important pest species and is mainly a foliage-feeder, compared to other pests in cotton that are fruit-feeders. Additionally, this species is an important model species for plant-insect interactions, which makes it easier to put data into a wider context.

We fed larvae of *S. littoralis* with cotyledons from the three selected cotton lines and recorded several performance parameters in order to test if the low gossypol content of ULGCS cotton could benefit the herbivore. Gossypol concentrations of the cotyledons were measured via a high-performance liquid chromatography (HPLC) to link changes in larval performance to changes in plant chemistry. In a second step, we analyzed the larval performance on the true leaves of the selected cotton lines. Larvae were fed with leaves from plants which were either undamaged, damaged by *S. littoralis* larvae, or induced by treatment with jasmonic acid. Additionally, we assessed the larval behavior under choice conditions to detect potential changes in attraction toward the different plants.

## Results

### Cotyledon assay

#### Terpenoid content

**Fig. 1:**
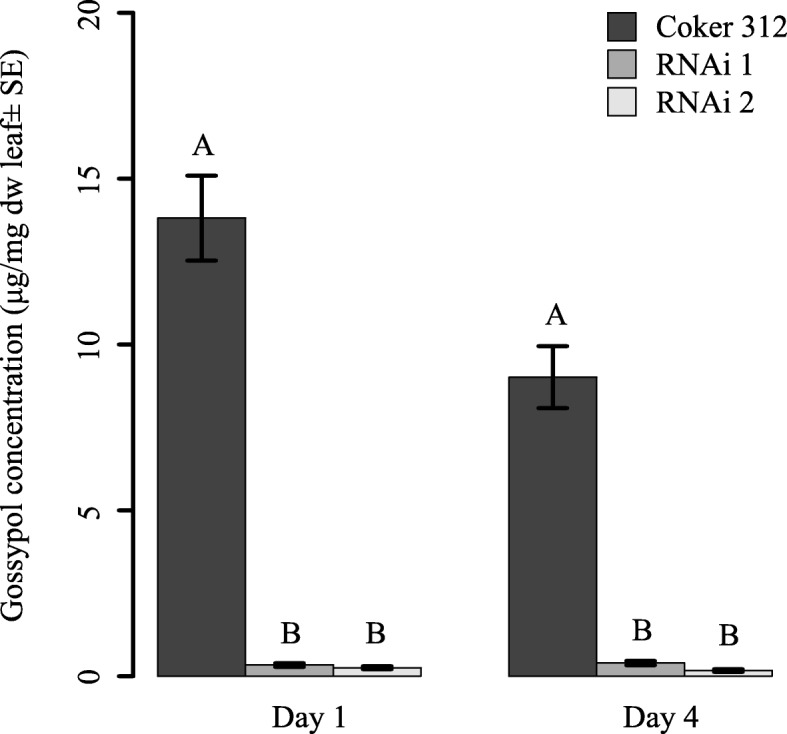
Concentration of gossypol in cotyledons as affected by plant type. Gossypol levels (μg/mg ± SE) in cotyledons from conventional cotton (Coker 312) and two ULGCS cotton lines (RNAi 1: 66-49B and RNAi 2: 66–274). One cotyledon was collected at the start of the feeding assay (day 1). The second cotyledon was collected on day four of the assay. Different capital letters over bars from the same sample day indicate a significant difference between plant types (Tukey HSD test), *n* = 32–36

Cotyledons contained gossypol, but no measurable amounts of hemigossypolone, heliocides H1 + H4, and heliocides H2 + H3. Gossypol concentrations were much lower in the two ULGCS cotton lines (day 1: F_2,78_ = 83.6, *p* < 0.001; day 4: F_2,81_ = 67.7; *p* < 0.001), and in some cases not detectable (RNAi 1: day 1: 28.6% of tested plants, day 4: 20.0% of tested plants; RNAi 2: day 1: 33.3% of tested plants, day 4: 38.9% of tested plants) when compared to non-transgenic near isoline (Coker 312) (Fig. 1). Gossypol concentrations in cotyledons from the non-transgenic plants declined significantly between the start of the assay (day 1) and the time point when the second cotyledon was harvested (day 4) (F_1,62_ = 9.2, *p* = 0.004) (Fig. 1).

#### Larval performance

When fed with cotyledons from all three cotton lines, no effect was visible on the survival of *S. littoralis* larvae during the seven day feeding period (Table 1). However, differences in larval weight were evident after four and seven days, with weights of the larvae being significantly lower on Coker 312 plants compared to the two ULGCS lines. After seven days, larvae had consumed significantly less material from the Coker 312 leaf discs compared to the two ULGCS lines (Table 1).

**Table 1:** Performance of *Spodoptera littoralis* larvae on cotyledons from conventional and ULGCS cotton. First instars were fed with cotyledon leaf discs from conventional cotton (Coker 312) and two ULGCS cotton lines (RNAi 1: 66-49B and RNAi 2: 66–274) for seven days. Survival, weight, and consumed leaf area were measured after four and seven days. Means within one column followed by different letters are significantly different (Tukey HSD test) (*n* = 30–32)

Cotton type	Four days			Seven days		
	Weight (mg ± SE)	Leaf area consumed (cm^2^ ± SE)	Survival (%)	Weight (mg ± SE)	Leaf area consumed (cm^2^ ± SE)	Survival (%)
Coker 312	1.8 ± 0.23 b	0.46 ± 0.083	59.4	28.9 ± 4.14 b	2.70 ± 0.35 b	59.4
RNAi 1	2.8 ± 0.32 a	0.51 ± 0.064	82.9	43.1 ± 4.35 a	4.74 ± 0.41 a	77.1
RNAi 2	3.3 ± 0.27 a	0.67 ± 0.079	72.7	49.8 ± 3.97 a	5.03 ± 0.36 a	69.4
GLM	F _2,71_ = 5.9; *p* = 0.004	F _2,69_ = 1.9; *p* = 0.160	χ^2^_2,100_ = 4.6; *p* = 0.100	F _2,68_ = 5.9; *p* = 0.004	F _2,68_ = 11.3; *p* < 0.001	χ^2^_2,100_ = 2.5; *p* = 0.290

### True leaf assay

#### Terpenoid content

Hemigossypolone concentration was affected by plant type and treatment but there was no significant interaction detected (Plant: F _2, 187_ = 5.3, *p* = 0.006; Treatment F _2, 185_ = 49.7, *p* < 0.001, Plant × Treatment F _4, 181_ = 1.0, *p* = 0.389). Gossypol concentration was affected by plant type and treatment but there was no significant interaction detected (Plant: F _2, 183_ = 12.0, *p* < 0.001; Treatment F _2, 181_ = 23.6, *p* < 0.001, Plant × Treatment F _4, 177_ = 1.0, *p* = 0.390). Heliocide 1 + 4 concentration was not affected by plant type, but by treatment. There was no significant interaction detected (Plant: F _2, 184_ = 2.67, *p* = 0.072; Treatment F _2, 182_ = 27.92, *p* < 0.001, Plant × Treatment F _4, 178_ = 0.85, *p* = 0.498).

**Fig. 2:**
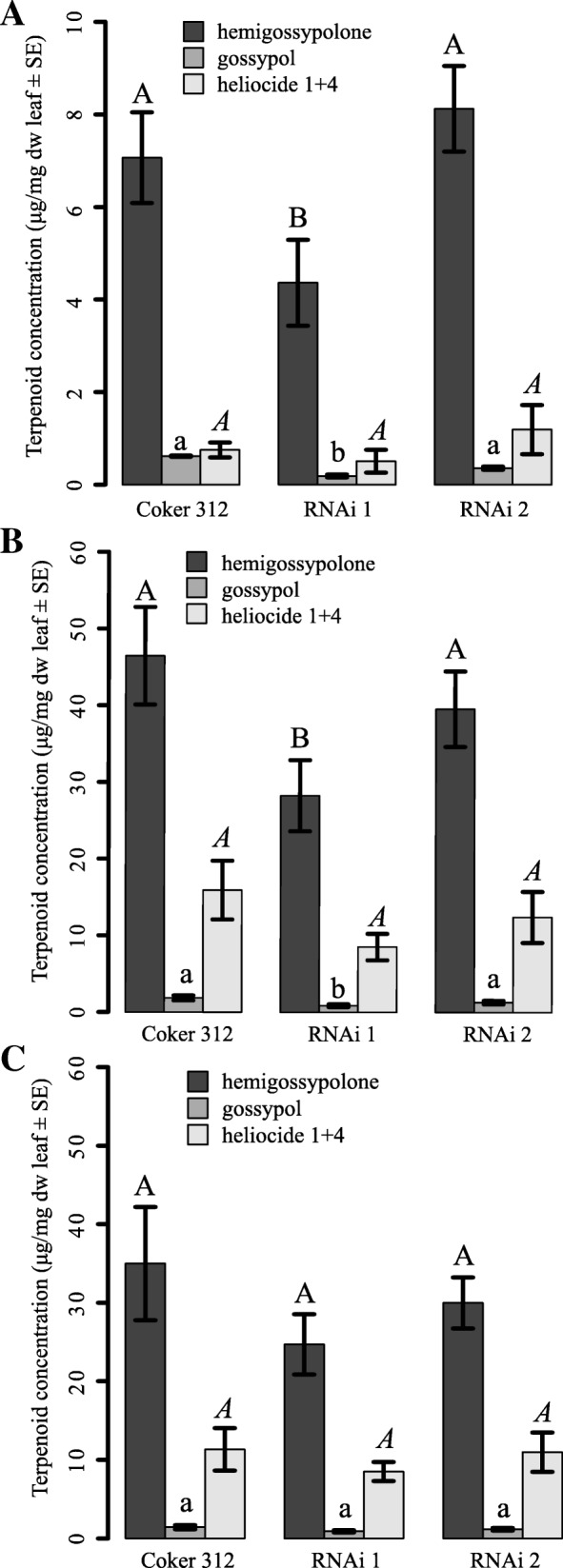
Impact of plant type and plant induction on concentration of terpenoids in cotton. Terpenoid levels (μg/mg ± SE) in the youngest leaves collected from conventional cotton (Coker 312) and two ULGCS cotton lines (RNAi 1: 66-49B and RNAi 2: 66–274). A) Cotton plants were untreated. B) Cotton plants had been exposed to three 2nd instar *S. littoralis*. C) Cotton plants were treated with jasmonic acid (4.8 μmol). Within induction treatments, different letters over the same chemical compound indicate a significantly difference between plant types (upper case letter: hemigossypolone; upper case letter italics: heliocide H1 + H4; lower case letter: gossypol) (Tukey HSD test), *n* = 20–25

The youngest leaves of untreated ULGCS and Coker 312 cotton contained significant amounts of gossypol, hemigossypolone, and heliocides H1 + H4. We were not able to verify the presence of the heliocides H2 + H3. The concentrations of hemigossypolone and gossypol were equal in Coker 312 and RNAi 2, but significantly lower in RNAi 1 (hemigossypolone: F_2,66_ = 4.95, *p* = 0.021; gossypol: F_2,63_ = 8.09, *p* < 0.001; Fig. 2a). Concentrations of heliocides H1 + H4 did not differ significantly among plant lines (F_2,64_ = 0.902, *p* = 0.411).

To induce the plants, they were infested with *S. littoralis* larvae before the start of the experiment. The plant type had no effect on the consumed leaf areas (Coker: 21.9 ± 2.42 cm^2^ ± SE; RNAi 1: 19.4 ± 2.34 cm^2^ ± SE; RNAi 2: 22.6 ± 2.97 cm^2^ ± SE; ANOVA: F_2,50_ = 0.521; *p* = 0.597). This leaf damage resulted in a strongly increased concentration of terpenoids in the youngest leaf compared to uninduced plants (Fig. 2b). Again, leaves from RNAi 1 contained a significantly lower concentration of gossypol and hemigossypolone than the remaining plant types (Fig. 2b) (hemigossypolone F_2,59_ = 3.185, *p* = 0.049, gossypol F_2,59_ = 5.306, *p* = 0.008), while no difference was detected for heliocides H1 + H4 (F_2,58_ = 1.639, *p* = 0.203).

Plants that were treated with jasmonic acid had an increased content of terpenoids compared to uninduced plants, but a lower concentration than plants infested with *S. littoralis* (Fig. 2c). However, no significant difference in content was found among the three plant types for any of the terpenoids analyzed (hemigossypolone: F_2,56_ = 1.042, *p* = 0.359; gossypol: F_2,55_ = 1.838, *p* = 0.169; H1 + H4: F_2,56_ = 0.536, *p* = 0.388) (Fig. 2c).

#### Larval performance

When *S. littoralis* were feedin on leaf discs from different cotton plants during the first seven days of their development, we found no strong impact of the factor plant type, but a strong difference between induced and uninduced plants. After four and seven days, larvae feeding on leaf discs from untreated plants were generally heavier and had consumed a larger leaf area compared to larvae fed discs from *S. littoralis*-infested or jasmonic acid-treated plants (Tables 2 and 3). The overall analyses revealed that plant type had no influence on the consumed leaf area and only an influence on larval weight after four days, but not after seven days. However, interactions between plant type and treatment were detectable in all cases. This is best explained by a better performance of larvae on ULGCS cotton lines that had been induced by *S. littoralis* feeding, compared to similarly treated Coker 312, as is evident in an increased larval weight and a larger leaf area consumed. These interactions were assessed with interaction plots, which can be found in the Additional file 1: Figs. S1-S4). Survival was not affected by either plant type or treatment, nor was there a significant interaction (Plant: χ^2^_2, 182_ = 0.33, *p* = 0.850; Treatment χ^2^_2, 180_ = 0.12, *p* = 0.940, Plant x Treatment χ^2^_2, 176_ = 0.85, *p* = 0.931).

**Table 2:** Performance of *Spodoptera littoralis* larvae on leaves from conventional and ULGCS cotton. First instars were fed with leaf discs from conventional cotton (Coker 312) and two ULGCS cotton lines (RNAi 1: 66-49B and RNAi 2: 66–274) for 7 days. Survival, weight, and consumed leaf area were measured after four and 7 days. Four larvae received leaf material from one plant and were pooled for analyses, 20 plants were tested per treatment (*n* = 20)

Cotton type	Four days			Seven days		
	Weight (mg ± SE)	Leaf area consumed (cm^2^ ± SE)	Survival (Number larvae ± SE)	Weight (mg ± SE)	Leaf area consumed (cm^2^ ± SE)	Survival (Number larvae ± SE)
Untreated cotton plants						
Coker 312	3.96 ± 0.260	0.90 ± 0.062	3.29 ± 0.171	36.64 ± 3.653	5.14 ± 0.435	3.19 ± 0.178
RNAi 1	4.14 ± 0.337	1.12 ± 0.094	3.27 ± 0.150	35.78 ± 3.101	5.33 ± 0.336	3.14 ± 0.190
RNAi 2	4.01 ± 0.218	0.94 ± 0.056	3.27 ± 0.199	34.04 ± 2.832	5.03 ± 0.267	3.14 ± 0.190
*Spodoptera littoralis* damaged cotton plants						
Coker 312	1.29 ± 0.164	0.40 ± 0.058	3.40 ± 0.240	6.38 ± 0.882	1.18 ± 0.158	3.15 ± 0.261
RNAi 1	2.05 ± 0.239	0.70 ± 0.081	3.40 ± 0.152	9.98 ± 1.726	1.82 ± 0.308	3.25 ± 0.160
RNAi 2	1.96 ± 0.246	0.64 ± 0.081	3.55 ± 0.135	12.24 ± 1.806	1.86 ± 0.254	3.25 ± 0.160
Jasmonic acid treated cotton plants						
Coker 312	2.37 ± 0.159	0.74 ± 0.058	3.70 ± 0.128	21.75 ± 2.585	3.38 ± 0.302	3.55 ± 0.135
RNAi 1	2.44 ± 0.169	0.80 ± 0.068	3.35 ± 0.196	19.94 ± 1.940	3.16 ± 0.315	3.30 ± 0.193
RNAi 2	2.72 ± 0.232	0.78 ± 0.069	3.05 ± 0.235	19.92 ± 2.565	3.22 ± 0.392	2.95 ± 0.211

**Table 3:** GLM results of performance assay. Effects of plant type (factor with three levels: Coker 312; RNAi 1 and RNAi 2) and treatment (factor with three levels: untreated, *S. littoralis* infested and jasmonic acid treated) and their interaction on weight and leaf area consumed by *S. littoralis* larvae in the performance assay with true leaves after four and seven days (*n* = 20)

		Plant	Treatment	Plant × Treatment
4 days	Leaf area consumed	F_2, 181_ = 1.88; *p* = 0.16	F_2, 179_ = 63.3; *p* < 0.001	F_4, 175_ = 3.19; *p* = 0.02
	Weight	F_2, 181_ = 4.72; *p* = 0.01	F_2, 179_ = 22.3; *p* < 0.001	F_4, 175_ = 2.82; *p* = 0.03
7 days	Leaf area consumed	F_2, 181_ = 0.01; *p* = 0.99	F_2, 179_ = 82.3; *p* < 0.001	F_4, 175_ = 3.55; *p* = 0.01
	Weight	F_2, 181_ = 0.21; *p* = 0.80	F_2, 179_ = 79.4; *p* < 0.001	F_4, 175_ = 2.50; *p* = 0.04

For a subset of larvae on untreated plants of the three cotton lines, the larvae were allowed to complete their development to the pupal stage. We found no significant differences among plant types for weight after 14 days (F_2, 47_ = 2.80; *p* = 0.07), development time (χ^2^_2, 39_ = 0.65; *p* = 0.72), pupation rate (χ^2^_2, 57_ = 0.48; *p* = 0.79), pupal weight (F_2, 37_ = 2.36; *p* = 0.11), or sex ratio (χ^2^_2, 39_ = 0.67; *p* = 0.72).

#### Preference assay

We conducted a series of choice assays to establish if ULGCS cotton types were more attractive for *S. littoralis* larvae than Coker 312 (Table 4). Larvae showed no preference for any of the plant types when the plants were treated equally (i.e., undamaged, damaged by caterpillars, or treated with jasmonic acid). The preference indices [calculated as: (*leaf area consumed Plant A – leaf area consumed Plant B)/total leaf area consumed*] for all of these comparison varied between − 0.30 and 0.12.

**Table 4:** Preference of *Spodoptora littoralis* larvae towards different cotton lines and different treatments. Third instars were given the choice between leaf discs from two different plants (Plant A vs Plant B). The leaf discs were either taken from conventional cotton (Coker 312) or two ULGCS cotton lines (RNAi 1: 66-49B and RNAi 2: 66–274). Additionally, plants were either untreated, damaged by larvae or treated with jasmonic acid. After 24 h the consumed leaf surface was measured. Preference indices were calculated as: *(leaf area consumed Plant A – leaf area consumed Plant B)/total leaf area consumed*. Three larvae received leaf discs from one plant pair and data were pooled for analyses, twenty plant pairs were tested per comparison (n = 20). Un = untreated; Spod = damage by *S. littoralis* larvae; JA = treated with jasmonic acid. The consumed leaf areas for Plant A and Plant B were compared using one-sided Wilcoxon signed-rank tests

Plant A	Plant B	Leaf area Plant A (cm^2^ ± SE)	Leaf area Plant B (cm^2^ ± SE)	Preference for Plant A (± SE)	Statistics
Coker Un	Coker Spod	1.216 ± 0.161	0.108 ± 0.030	0.76 ± 0.062	V = 209; *p* < 0.001
Coker Un	Coker JA	0.998 ± 0.151	0.334 ± 0.062	0.39 ± 0.119	V = 181; *p* = 0.003
RNAi 1 Un	RNAi 1 Spod	1.082 ± 0.137	0.324 ± 0.077	0.57 ± 0.079	V = 204; *p* = 0.001
RNAi 1 Un	RNAi 1 JA	1.191 ± 0.198	0.461 ± 0.098	0.39 ± 0.105	V = 186; *p* = 0.003
Coker Un	RNAi 1 Un	0.628 ± 0.086	0.709 ± 0.145	0.12 ± 0.135	V = 128; *p* = 0.737
Coker Spod	RNAi 1 Spod	0.549 ± 0.156	0.652 ± 0.106	− 0.28 ± 0.145	V = 59; *p* = 0.926
Coker JA	RNAi 1 JA	0.391 ± 0.102	0.760 ± 0.126	− 0.30 ± 0.124	V = 43; *p* = 0.120
RNAi 2 Un	RNAi 2 Spod	1.291 ± 0.167	0.169 ± 0.053	0.69 ± 0.090	V = 201; *p* < 0.001
RNAi 2 Un	RNAi 2 JA	1.017 ± 0.145	0.291 ± 0.068	0.52 ± 0.100	V = 195; *p* < 0.001
Coker Un	RNAi 2 Un	0.706 ± 0.130	0.836 ± 0.106	− 0.18 ± 0.096	V = 50; *p* = 0.120
Coker Spod	RNAi 2 Spod	0.415 ± 0.088	0.612 ± 0.108	− 0.18 ± 0.129	V = 72; *p* = 0.461
Coker JA	RNAi 2 JA	0.664 ± 0.123	0.598 ± 0.095	0.02 ± 0.104	V = 108; *p* = 0.926

Additional assays were performed to assess the response of larvae when given a choice between uninduced and induced (both by *S. littoralis* and jasmonic acid) leaves from the same plant type (Table 4). In these assays, we found a preference for uninduced plant material regardless of plant type or type of induction. When larvae were allowed to choose between undamaged and *S. littoralis* damaged plants, preference indices varied between 0.57 and 0.76 (Table 4). Statistical comparison of the indices showed that this response was similar in the two transgenic lines compared to Coker 312 (Coker vs. RNAi 1: V = 280; *p* = 0.130; Coker vs. RNAi 2: V = 222; *p* = 0.665). Similar results were obtained in the comparisons between undamaged and jasmonic acid treated plants where the preference indices varied between 0.39 and 0.52 (Table 4). A comparison of the indices revealed no difference between the two transgenic lines and Coker 312 (Coker vs. RNAi 1: V = 217; *p* = 0.665; Coker vs. RNAi 2: V = 175; *p* = 0.665).

## Discussion

During this study, we assessed whether two ULGCS cotton lines have a reduced insect-resistance compared to conventional, non-transformed cotton plants.

The comparison of terpenoid (hemigossypolone, gossypol, heliocide H1 + H4) concentrations in true leaves from Coker 312 and the two ULGCS lines revealed significantly lower concentrations in the leaves of one of the two ULGCS lines (RNAi 1). The pattern was evident in uninduced as well as in induced plants. This, however, does not seem to be a general side-effect of reduced δ-cadinene synthease activity that has proven to be completely seed-specific [9, 10, 13]. Also, terpenoid production in the second line (RNAi 2) was similar compared to the conventional control plants and was induced in both lines in response to caterpillar-damage or treatment with jasmonic acid. The differences between the two ULGCS cotton lines might be due to the tissue culture and very long regeneration process resulting in somaclonal variation. Secondary plant metabolite concentrations can vary among different cultivars, varieties or genotypes which is also true for cotton [14]. Therefore, some variation among different lines of ULGCS and conventional cotton can be expected. Such variation, however, should be considered when selecting the most suitable transformation lines for further development. Certain differences in terpenoid concentrations among different RNAi lines were reported before [11]. Our line RNAi 2 has recently been approved by US regulators as event TAM66274 [10].

As expected, feeding bioassays with *S. littoralis* revealed that their performance (weight increase, consumed leaf area) was reduced on induced cotton plants. When plants were undamaged or had been treated with jasmonic acid, no difference in *S. littoralis* performance among the three cotton lines was observed. However, when plants had previously been damaged by caterpillars, *S. littorals* performance was significantly better on the two ULGCS lines as compared to the Coker 312 control plants. The fact that terpenoid concentrations were lower in RNAi 1 plants as compared to RNAi 2 plants, had no effect on the caterpillar performance. The effects might thus be due to some other phytochemical changes in the plant as a response to caterpillar damage [15].

Behavioral choice experiments revealed no preference by *S. littoralis* larvae for any of the three cotton lines. However, leaf discs from uninduced plants were always preferred over those from induced plants.

The picture is clearer for the cotyledons of ULGCS plants as they were significantly more susceptible to *S. littoralis* damage. A very low gossypol content in ULGCS cotyledons has been reported before [11]. Most likely, the cotyledons receive their terpenoids from the seed itself, which would explain why ULGCS lines contain low amounts of terpenoids [11]. Although *S. littoralis* is not a relevant pest of cotton seedlings, other herbivores, e.g. thrips or flea beetles, can cause significant damage during this stage [16]. Therefore, pest management strategies for ULGCS cotton need to take this potential vulnerability into account to prevent yield losses due to insect damage in this very early growth stage. This might not be a concern in production regions where neonicotinoid seed-treatments for cotton is common [17, 18].

Plants can react to herbivore damage by either tolerating the damage or actively defending themselves [19]. Both strategies are costly either due to association costs or biomass loss [20, 21], but the costs in both cases are outweighed when reproductive success is maintained [22]. However, under agronomical situations, high yield is economically important rather than successful reproduction. Therefore, plants are often grown under quasi pest free conditions, where herbivores are removed for example by using pesticides [1]. In recent years, a goal has been to reduce the application of pesticides by strengthening herbivore resistance traits. In cotton, inducible terpenoids play an important role and plants that do not produce these terpenoids are less resistant against herbivores and pathogens [6, 23], therefore, maintaining this resistance trait is also highly important for the long-term success of ULGCS cotton. Herbivore damage in cotton plants increases the abundance of terpenoids and other relevant resistance traits, which has a negative impact on herbivores [24]. While previous studies have looked into the terpenoid-based resistance mechanisms of ULGCS cotton, they have not addressed the impact of the trait on plant-insect interactions. A previous study tested the disease resistance of ULGCS seedlings and did not find any difference in their susceptibility to the fungus *R. solani* [11]. Our study complements this previous work by expanding resistance research in ULGCS cotton into insect resistance mechanisms. While cotton terpenoids play a role in both herbivore and pathogen resistance, these are thought to be regulated differently and therefore the transformation could affect these pathways differently. Our research indicates that the terpenoid-based insect resistance remains intact in the ULGCS lines tested, at least when true leaves are considered.

As our study was conducted under controlled laboratory conditions it is important to validate the results in the field with varying climate conditions and under the pressure of multiple herbivores belonging to different feeding guilds. A study by Palle et al. [13] found that terpenoid concentrations in ULGCS (including the RNAi 1 line used in the present study) and conventional plants were similar under field conditions. While we found a reduced terpenoid (hemigossypolone, gossypol) concentration in the RNAi 1 line, these differences in the results are possibly explained by different responses of the plants under field and greenhouse conditions. Overall, 7 years of field studies at Texas A&M have not indicated any difference in pest susceptibility between the RNAi-transgenic ULGCS lines and the untransformed plants [10, 13]. In addition, eight different, multi-state, regulatory field trials conducted over 2 years across the cotton belt in the U.S. did not show a higher degree of pest-susceptibility of the RNAi lines compared to the non-transgenic control [25].

While we could not find a strong impact of ULGCS cotton on the direct, terpenoid-based insect resistance traits, indirect resistance traits were not considered in the present study. The latter are important because they attract/arrest natural enemies of the cotton herbivores to infected plants [19]. A key part in this recruitment is the release of herbivore-induced volatiles (HIVPs). This system is well studied in cotton and the volatile blend of cotton is rich in small terpenes such as δ-cadinene [26]. As ULGCS cotton suppresses the production of δ-cadinene synthase in seeds, this could affect the composition of the plants volatile blend, however, only during the very early stages of the growth.

## Conclusions

We found that the terpenoid-based constitutive and induced insect resistance in ULGCS cotton lines is little affected by the genetic transformation when compared to conventional cotton. At the same time, these plants have a greatly reduced amount of terpenoids in the seeds as intended. As a consequence, gossypol content and thus resistance in cotyledons is weaker and *S. littoralis* larvae perform better on ULGCS cotyledons than on conventional ones. Therefore, ULGCS cotton plants might be more susceptible to insect damage during the early stages after germination and may require additional chemical protection, as it is routinely practiced by farmers in many cotton-growing countries.

## Methods

### Insects

Eggs of *S. littoralis* were provided by Syngenta and sent on a weekly basis from Stein, Switzerland. Larvae were kept at 25 °C, 70% RH, a 16:8 day:night light cycle, and fed with Heliothis Stonefly Diet (Ward Science, Rochester NY, USA) until they reached the desired stage.

### Plants

Plants of two ULGCS lines (RNAi 1: line 66-49B; RNAi 2: line 66–274) were used (see [11] for a description of the lines). Additionally, non-transgenic Coker 312 plants, the paternal (untransformed) cultivar which was used to create the two ULGCSs lines, were serving as a control (termed “conventional cotton”). All plant material has been provided by Keerti S. Rathore (Texas A & M University, USA). Plants were initially grown in small pots in a climate chamber with 25 °C, 70% RH, and a 16:8 day:night light cycle. After 10 days, the plants were transferred to 3 l plastic pots, containing humus-rich soil enriched with 15 mg of the slow release fertilizer Manna Cote 4 M (Wilhelm Haug GmbH, Ammerbuch, Germany). They were moved to a climate controlled greenhouse and grown under the same conditions as in the climate chamber. After 4 weeks the plants were once fertilized with 10 N: 10P: 8 K at 10 ml/L. In the greenhouse, plants were enclosed in gauze cages (height, 71 cm; diameter, 35 cm; mesh-width, 0.264 mm) to protect them from glasshouse pests. Plants needed for experiments at the cotyledon stage were used 10 days after sowing, while plants used for the true leave assays were used after 4–5 weeks.

### Plant induction

Plants that had four fully-developed true leaves and did not show any signs of damage caused by herbivores or diseases were treated in one of three different ways: (i) plants were left untreated (control), (ii) plants were induced with 4.8 μmol of jasmonic acid (Sigma-Aldrich; MO, USA), or (iii) plants were induced by releasing three pre-weighted 2nd instar *S. littoralis* larvae on the second or third true leaf (counted from the bottom of the plant). The larvae were contained on the leaf using an organdy cloth bag. Jasmonic acid was applied in 1 ml of an ethanol: water solution (20 μl ethanol: 980 μl of water). The solution was directly applied to the stem of the plant, thereby allowing uptake of jasmonic acid by above- and below-ground tissue. Hagenbucher et al. [27] and Eisenring et al. [28] showed that the application of jasmonic acid induces the terpenoid-based defence in cotton. After 1 week the plants were used for the different experiments. Damage and performance of *S. littoralis* were assessed by weighing the surviving larvae and measuring the feeding damage by scanning the leaves and measuring the consumed leaf area using the software Image J 1.48 (NIH, USA; [29]).

### Performance assay with cotyledons

This feeding experiment was conducted to assess the performance of *S. littoralis* larvae on cotyledons of the different cotton lines. Plants were grown as described above but remained in the climate chamber. The plants were used 10 days after sowing. All plants remained untreated (no induction). From these cotyledons, 1 cm leaf discs were taken and transferred to the wells of 128-well plastic bioassay trays which were sealed with 16 cell tray covers (Bio-Serv; Flemington, NJ, USA). The remaining leaf material was stored at − 80 °C and used for terpenoid quantification. The bottom of each well was covered with a 1% agar-gel to keep the leaf discs moist. Agar was used since it completely binds water and therefore prevents the formation of small droplets that could be hazardous for the extremely sensitive neonates. On each disc, a single neonate *S. littoralis* was placed. After 4 days the larvae were transferred to a 3 cm diameter Petri-dish, containing a new 3 cm leaf disc from the second cotyledon of the same plant. The bottom of the Petri-dish was covered with plaster (Quickmix; Quickmix-Gruppe, Osnabrück, Germany) to provide moisture. Plaster was used for older larvae as it has the advantage that it cannot be consumed and does not provide energy compared to agar. After a total of 7 days, the assay was terminated. The following data were recorded after four and 7 days: larval survival, weight, larval stage, and consumed leaf area using Image J 1.48. A total of 30–32 larvae was tested per cotton line.

### Performance assay with true leaves

This feeding experiment was conducted to assess if the performance of *S. littoralis* is affected by transformation or induction. Therefore, the youngest fully developed leaf from plants of all three lines and all three induction treatments were collected to be used in a feeding assay.

From these leaves, 1 cm leaf discs were taken and transferred to the wells of 128-well plastic bioassay trays which were sealed with 16 cell tray covers (Bio-Serv). The remaining leaf material was stored at − 80 °C and used for terpenoid quantification. The bottom of each well was covered with a 1% agar-gel to keep the leaf discs moist, as described above. On each leaf disc, a single neonate *S. littoralis* was placed. After 4 days, the larvae were transferred to a 3 cm diameter Petri-dish, containing a new 3 cm leaf disc from the same plant, retrieved from the actual youngest fully-developed leaf at that time. The bottom of the Petri-dish was covered with plaster (Quickmix) to provide moisture. After three additional days, the experiment was terminated. The following data were recorded after 4 and 7 days: larval survival, weight, and the leaf area consumed. Each plant used in this experiment was used to feed four different larvae. A total of 20 plants were tested in this way per plant type and treatment. Data for larvae feeding on leaves from one plant were pooled to avoid pseudo-replication, resulting in 20 replications (with a total of 80 larvae tested per treatment). Data recorded for larvae that died during the experiment were not included in the analysis of sublethal parameters.

For a subset of larvae on untreated plants of the three plant lines, the larvae were allowed to complete development to the pupal stage. From each plant, one larva surviving the first 7 days was selected randomly and fed with leaf discs from the same plant until pupation or death. Survival, development time, sex ratio, weight after 14 days and pupal weight (3 days after pupation) were recorded. This part of the experiment was not conducted with induced plants, because high food consumption of late instar *S. littoralis* makes it difficult to supply the larvae with leaves of consistent quality (degree of induction) over the entire duration of the experiment.

### Preference assay

This experiment was conducted to establish if transformation and terpenoid induction affect the feeding preference of *S. littoralis* under choice conditions. At the onset of the experiment, the youngest fully developed leaf of each plant was harvested and leaf-discs of 2 cm diameter were cut. Leaf-discs from two different plants were placed on opposite sides of a Petri-dish (diameter 9 cm) and a single 3rd instar *S. littoralis* larva was released in the middle. The Petri-dishes were then moved to a climate cabinet (25 °C, 70% RH, and a 16:8 day:night light cycle). After 24 h the consumed leaf surface was quantified for both leaf discs by scanning the discs and measuring the consumed leaf area using Image J 1.48. From this dataset, a preference index was calculated: (leaf area consumed Plant A – leaf area consumed Plant B)/total leaf area consumed. Discs from one plant-pair were used to test three different larvae, in total 20 plant-pairs were tested for each comparison. The leaf material that remained after cutting the three discs was stored at − 80 °C and used for terpenoid quantification. To avoid pseudo-replications, data for larvae tested with leaf discs from the same plant were pooled, resulting in 20 replications (with a total of 60 larvae tested per treatment).

### Terpenoid quantification

Plant material collected in the different experiments was analyzed via HPLC system (1090 Series 219 II, Hewlett-Packard, Palo Alto, USA; column: Varian Polaris Amide C-18 column, 150 × 2.0 mm, 3 μm, equipped with a precolumn C18, 4 × 3.0 mm, Supelco Security Guard System). HPLC analyses followed the methodology described by Hagenbucher et al. [27], which is capable of quantifying gossypol, hemigossypolone, heliocides H1 + H4, and heliocides H2 + H3.

### Statistics

The cotyledon assay was analyzed using a GLM with plant type as independent variable and the dependent variables survival rate (quasipoisson, due to overdispersion), weight (gamma distribution), and consumed leaf area (gamma distribution). Means were subsequently separated using Tukey’s HSD test.

The performance assays with true leaves were analyzed using a GLM with plant type, induction treatment, and the interaction of both as independent variables. Dependent variables were survival rate (quasipoisson, due to overdispersion), weight (gamma distribution), and consumed leaf area (gamma distribution). Means were subsequently separated using Tukey’s HSD test. The data for larvae that were fed during their entire development time with the three cotton types was analyzed using a GLM. Therefore, we used plant type as independent variable while pupal weight (gamma distribution), development time (poisson distribution), weight after 14 days (gamma distribution) and sex ratio (binomial distribution) were used as the dependent variables.

To analyze the preference (indicated as consumed leaf area) of *S. larvae* for one of two leaf discs in the choice experiments, one-sided Wilcoxon signed-rank tests were conducted. The preference indices among different choice tests were subsequently compared using two-sided Wilcoxon signed-rank tests.

The terpenoid content of mature cotton leaves were analyzed using a GLM with plant type, induction treatment, and the interaction of both as independent variables. Dependent variables were gossypol concentration (normal distribution), hemigossypolone concentration (normal distribution), and Heliocide 1 + 4 concentration (normal distribution). For an in depth analyses the concentrations of the terpenoids were analyzed with a GLM (assuming normal distribution) for each of three treatments (untreated, *S. littoralis* infested, and jasmonic acid-treated). These analyses were done separate for each of the three terpenoid classes. Means were subsequently separated using Tukey’s HSD test.

All data were analyzed using R3.1.0 statistical software (https://www.r-project.org/).

## Additional file

Additional file 1: **Figure S1.** Interaction plot for leaf area consumed by *S. littoralis* larvae after 4 days. **Figure S2.** Interaction plot for leaf area consumed by *S. littoralis* larvae after 7 days. **Figure S3.** Interaction plot for weight of *S. littoralis* larvae after 4 days. **Figure S4.** Interaction plot for weight of *S. littoralis* larvae after 7 days. (DOCX 3984 kb)

## Data Availability

The authors confirm that all data underlying the findings are fully available without restriction. All relevant data are available on the figshare Data Repository (http://dx.doi.org/10.6084/m9.figshare.8329760).

## Abbreviations

HPLC: High-performance liquid chromatography
RNAi 1: ULGCS cotton line 66-49B
RNAi 2: ULGCS cotton line 66–274
RNAi: RNA interference
ULGCS: Ultra-low gossypol cottonseed
