# Supplementary material for: Constitutive and induced insect resistance in RNAi-mediated ultra-low gossypol cottonseed cotton
Source: BMC Plant Biol. 2019 Jul 18;19:322. doi: 10.1186/s12870-019-1921-9 (PMC6639952; doi:10.1186/s12870-019-1921-9)
Supplement: Supplementary file 1 — Figure S1. Interaction plot for leaf area consumed by S. littoralis larvae after 4 days. Figure S2. Interaction plot for leaf area consumed by S. littoralis larvae after 7 days. Figure S3. Interaction plot for weight of S. littoralis larvae after 4 days. Figure S4. Interaction plot for weight of S. littoralis larvae after 7 days. (DOCX 3984 kb) [file 12870_2019_1921_MOESM1_ESM.docx]

**Constitutive and induced insect resistance in** **RNAi-mediated ultra-low gossypol cottonseed cotton**

Steffen Hagenbucher^1^, Michael Eisenring^1^, Michael Meissle^1^, Keerti S Rathore^2^ and Jörg Romeis^1,^*

^1^ *Agroscope, Research Division Agroecology and Environment, Reckenholzstrasse 191, 8046 Zürich, Switzerland;* ^2^ *Institute for Plant Genomics & Biotechnology, Department of Soil & Crop Sciences, Texas A&M University, College Station, TX, USA*


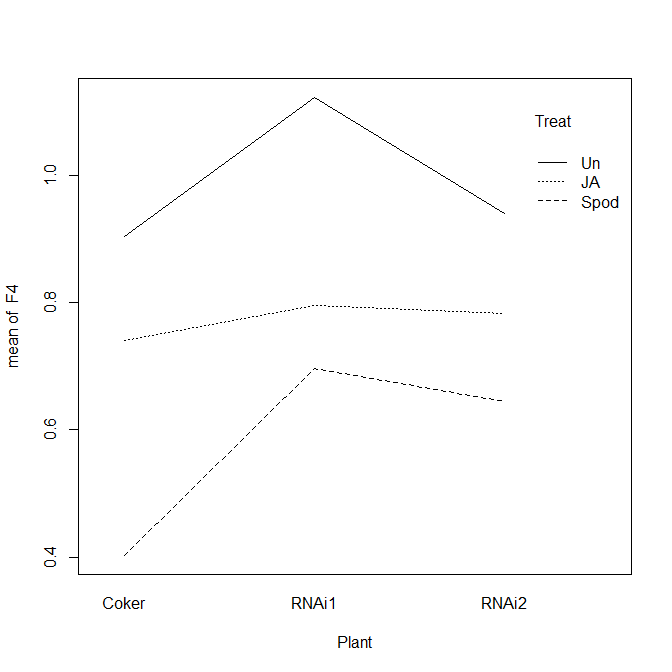
**Corresponding Author: Jörg Romeis; joerg.romeis@agroscope.admin.ch*

**Fig. S1** Interaction plot for leaf area consumed by *S. littoralis* larvae after four days. The x-axis displays the categorical variable plant type: either conventional cotton (Coker 312) or two ULGCS cotton lines (RNAi 1: 66-49B and RNAi 2: 66-274). The y-axis displays the variable consumed leaf area after four days (mean of F4).


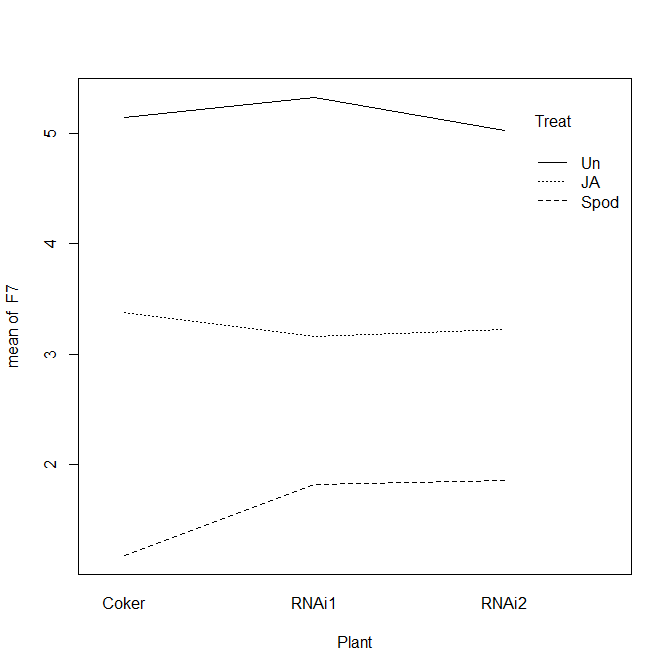


**Fig. S2** Interaction plot for leaf area consumed by *S. littoralis* larvae after seven days. The x-axis displays the categorical variable plant type: either conventional cotton (Coker 312) or two ULGCS cotton lines (RNAi 1: 66-49B and RNAi 2: 66-274). The y-axis displays the variable consumed leaf area after seven days (mean of F7).


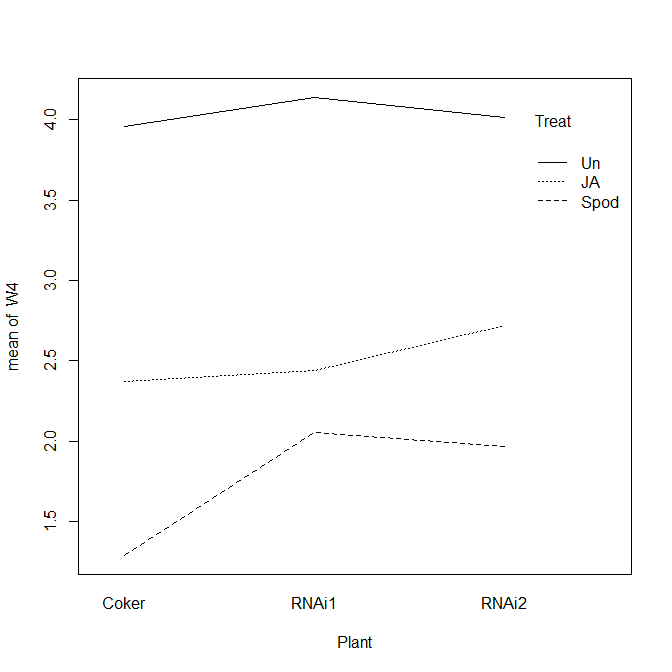


**Fig. S3** Interaction plot for weight of *S. littoralis* larvae after four days. The x-axis displays the categorical variable plant type: either conventional cotton (Coker 312) or two ULGCS cotton lines (RNAi 1: 66-49B and RNAi 2: 66-274). The y-axis displays the variable larval weight after four days (mean of W4).


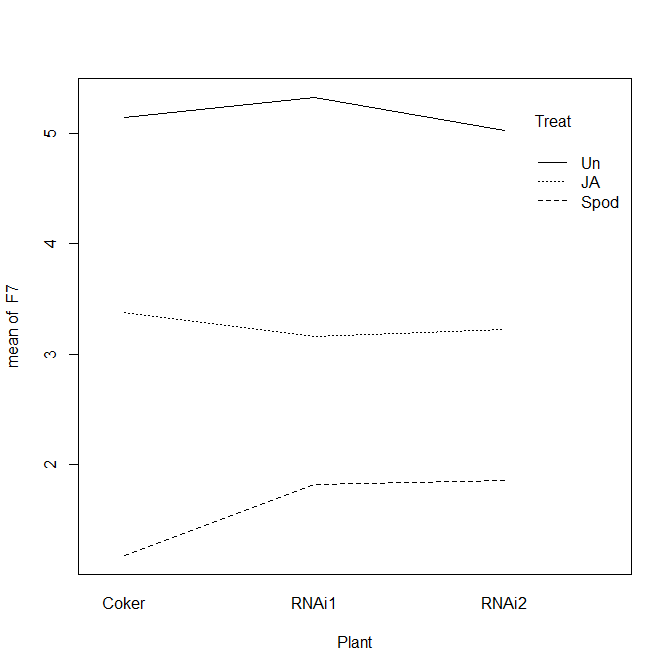


**Fig S4** Interaction plot for weight of *S. littoralis* larvae after seven days. The x-axis displays the categorical variable plant type: either conventional cotton (Coker 312) or two ULGCS cotton lines (RNAi 1: 66-49B and RNAi 2: 66-274). The y-axis displays the variable larval weight after seven days (mean of W7).
